# Circulating Tumor DNA and microRNA–Based Liquid Biopsy for Longitudinal Treatment Monitoring and Recurrence Prediction in Pediatric Sarcomas: A Prospective Cohort Study

**DOI:** 10.32604/or.2026.078833

**Published:** 2026-05-21

**Authors:** Maher Kurdi, Amber Hassan, Bashar Reda, Anas Nooh, Mohammed Alsobaie, Alaa Alkhotani, Dahlia S. Mirdad, Majid Almansouri, Khalid Khashoggi, Manal Halwani, Motaz Fadul, Humaira Waseem, Siti S. Maidin, Muhammed Imtiaz Farid

**Affiliations:** 1 Department of Pathology, Faculty of Medicine, King Abdulaziz University, Rabigh, Saudi Arabia; 2 Department of Neurogenetics, Istituto Neurologico Nazionale a Carattere Scientifico, IRCCS, Pavia, Italy; 3 Department of Orthopedic Surgery, Faculty of Medicine, King Abdulaziz University, Jeddah, Saudi Arabia; 4 Department of Surgery, Doctor Suliman Fakeeh Hospital, Jeddah, Saudi Arabia; 5 Department of Pathology, College of Medicine, Umm Al-Qura University, Makkah, Saudi Arabia; 6 Department of Basic Medical Sciences, College of Medicine, University of Jeddah, Jeddah, Saudi Arabia; 7 Department of Clinical Biochemistry, Faculty of Medicine, King Abdulaziz University, Jeddah, Saudi Arabia; 8 Department of Radiology, Faculty of Medicine, King Abdulaziz University, Jeddah, Saudi Arabia; 9 Department of Emergency Medicine, Faculty of Medicine, King Abdulaziz University, Jeddah, Saudi Arabia; 10 Department of Data Science, INTI International University, Nilai, Malaysia; 11 Department of Quality Enhancement Cell, Fatima Jinnah Medical University, Lahore, Pakistan; 12 Primary & Secondary Health care Department, Maryam Nawaz Health Center, Pakpattan, Pakistan

**Keywords:** Pediatric sarcoma, liquid biopsy, circulating tumor DNA, microRNA, treatment monitoring, longitudinal surveillance

## Abstract

**Background:** Pediatric sarcomas are aggressive malignancies characterized by marked biological heterogeneity and a high risk of relapse. Standard surveillance relies on imaging and invasive biopsies, which may fail to detect early molecular disease. Liquid biopsy using circulating tumor DNA (ctDNA) and microRNAs offers a minimally invasive strategy for longitudinal monitoring. This study aimed to evaluate dynamic changes in ctDNA and circulating microRNAs during treatment and examined their associations with treatment response, disease recurrence, and survival outcomes. **Methods:** This prospective cohort study included 100 pediatric patients with histologically confirmed sarcomas. Serial peripheral blood samples were collected at diagnosis, during treatment, at treatment completion, and during follow-up. Plasma-derived ctDNA and miRNAs were quantified using droplet digital Polymerase Chain Reaction (PCR) and quantitative Reverse Transcriptase-PCR (qRT-PCR). Associations between biomarker levels, clinical and radiologic response, recurrence, and survival outcomes were analysed using correlation analyses. **Results:** The mean age at diagnosis was 10.5 ± 4.9 years, with a male predominance (59.0%). The most common tumor subtype was Ewing sarcoma (49.0%), followed by rhabdomyosarcoma (27.0%) and osteosarcoma (24.0%). Both ctDNA and circulating miRNA concentration declined significantly from baseline to the end of treatment (*p* < 0.001 for both). End-of-treatment biomarker levels showed weak and insignificant correlations with clinical response, radiologic response, and survival outcomes (all *p* > 0.05). In multivariable analysis, disease recurrence status was strongly associated with survival, as expected; however, circulating biomarker levels did not independently predict outcome (Odds ratio (OR) = 0.04, 95% Confidence Interval (CI): 0.01–0.15; *p* < 0.001). **Conclusions:** ctDNA and microRNA levels showed dynamic, treatment-related changes in pediatric sarcomas, reflecting therapeutic response at the population level. However, single end-of-treatment measurements were not predictive of outcomes. These findings support liquid biopsy for longitudinal monitoring rather than static prognostication and emphasize the need for prospective validation of serial biomarker approaches.

## 1 Introduction

Pediatric cancer remains a leading cause of disease-related mortality in children worldwide, despite substantial advances in multimodal therapy, risk stratification, and supportive care [1]. Among childhood malignancies, pediatric sarcomas including Ewing sarcoma, osteosarcoma, and rhabdomyosarcoma represent a biologically heterogeneous group of aggressive solid tumors with a persistently high risk of relapse and metastatic progression [2,3,4,5]. Although survival outcomes have improved for selected risk groups, disease recurrence continues to be the principal cause of cancer-related mortality in pediatric sarcoma patients [6,7]. Early and sensitive detection of treatment response and impending relapse, therefore, remains a critical unmet clinical need. Current surveillance strategies in pediatric sarcomas rely primarily on radiologic imaging and, when clinically indicated, invasive tissue biopsies [8]. While imaging plays an essential role in disease assessment, it may lack sensitivity for detecting early molecular relapse or minimal residual disease (MRD), particularly when tumor burden is low [9,10]. Moreover, repeated tissue sampling is often impractical or ethically challenging in children. These limitations emphasize the need for minimally invasive approaches that provide real-time insight into tumor dynamics, clonal evolution, treatment resistance, and early disease recurrence.

Ewing sarcoma, osteosarcoma, and rhabdomyosarcoma differ substantially in epidemiology and clinical behavior. Osteosarcoma most commonly arises in the metaphyses of long bones and frequently metastasizes to the lung, with outcomes strongly influenced by metastatic status at diagnosis [11]. Ewing sarcoma is characterized by recurrent gene fusions and often presents in bone or soft tissue, with relapse and metastatic disease remaining major determinants of survival [12,13]. Rhabdomyosarcoma comprises biologically distinct subgroups with variable anatomical distribution and prognosis. These differences underscore the need for minimally invasive biomarkers that can support subtype-aware longitudinal monitoring [14].

The concept of MRD detection has transformed clinical decision-making in haematological malignancies, enabling molecular monitoring to support risk-adapted therapy and improved outcome prediction [15]. However, in solid tumors, particularly pediatric sarcomas, standardised MRD assessment remains elusive due to tumor heterogeneity, anatomical constraints, and the absence of validated molecular markers in peripheral blood [16]. Translating the principles of molecular disease monitoring from hematologic cancers to pediatric solid tumours, therefore, requires novel and robust biomarker strategies.

Recent technological advances have enabled the detection of tumor-derived nucleic acids and cellular components in biofluids, giving rise to the field of liquid biopsy [15,17]. The considerable biological diversity and overlapping clinical features among bone tumors often complicate accurate diagnosis and, consequently, the selection of optimal personalized treatment strategies. Histologically similar lesions may exhibit markedly different molecular profiles and clinical behaviors, underscoring the importance of identifying tumor-specific genetic alterations to refine diagnostic precision in challenging cases [18]. Conventional cancer management relies heavily on tissue biopsy and surgical sampling, which remain the diagnostic gold standard for most malignancies [19]. While highly informative, these procedures are invasive and may not be feasible for repeated monitoring, particularly in pediatric patients or in anatomically complex sites [20]. Moreover, tumor heterogeneity poses a significant limitation, as distinct clonal populations can exist within different regions of the same tumor, meaning a single biopsy may not capture the full molecular landscape of the disease [21].

In contrast, liquid biopsy represents a minimally invasive approach capable of detecting tumor-associated molecular alterations in biological fluids such as blood, cerebrospinal fluid, pleural effusion, saliva, and urine [22]. Because circulating tumor-derived material may originate from both primary and metastatic sites, liquid biopsy has the potential to provide a more comprehensive and dynamic representation of tumor burden [23]. Importantly, it enables serial sampling over the course of therapy, facilitating real-time monitoring of disease evolution, treatment response, and early recurrence.

Circulating tumor DNA (ctDNA) and circulating microRNAs (miRNAs) have emerged as particularly promising biomarkers, as they reflect tumor-specific genetic and epigenetic alterations and can be repeatedly sampled with minimal patient burden [24,25]. ctDNA analysis allows real-time assessment of tumor burden and genetic evolution. At the same time, miRNAs, owing to their stability in plasma, may provide complementary information on tumor biology, treatment response, and metastatic potential [26,27].

In adult oncology, ctDNA-based liquid biopsy has demonstrated clinical utility for treatment monitoring, prognostication, and early relapse detection across multiple solid tumor types [28]. In contrast, the application of liquid biopsy in pediatric cancers remains comparatively underexplored.

Fundamental biological differences between pediatric and adult malignancies including lower mutational burden, higher prevalence of structural variants and gene fusions, and distinct tumor kinetics pose unique analytical and clinical challenges [29]. Additionally, pediatric blood sampling volumes are inherently limited, potentially affecting assay sensitivity, particularly in the context of low circulating tumor fractions [21]. Pediatric sarcomas present both challenges and opportunities for the development of liquid biopsy approaches. Many sarcomas are characterised by recurrent chromosomal translocations and dysregulated miRNA expression profiles, making them theoretically well-suited for targeted ctDNA and miRNA-based detection strategies [29]. However, the clinical validity and prognostic relevance of longitudinal ctDNA and miRNA monitoring during therapy and follow-up have not yet been systematically established in pediatric sarcoma cohorts [30].

Against this background, the present prospective cohort study aims to evaluate ctDNA- and miRNA-based liquid biopsies as minimally invasive tools for longitudinal treatment monitoring and recurrence prediction in pediatric sarcomas. By correlating biomarker dynamics with clinical outcomes, treatment response, and survival, this study seeks to generate clinically actionable evidence to support the integration of liquid biopsy approaches into routine pediatric sarcoma care.

## 2 Material and Methods

### 2.1 Study Design and Selection Criteria

This prospective longitudinal cohort study was conducted between November 2024 and December 2025. Ethical approval was obtained from the Ethics Review Committee of Fatima Jinnah Medical University and in a collaboration with scientific contributors from several Universities and Institutions including King Abdulaziz University, Saudi Arabia, and ITT International University, Malaysia and Mondino Foundation, Italy (Reference No. 198-Synopsis/Pediatrics-III/FJ/ERC; approved 11 November 2024). The study was conducted in accordance with the Declaration of Helsinki. Written informed consent was obtained from the legal guardians of all participating children prior to enrollment.

This study is reported in accordance with the STROBE guidelines for observational cohort studies. Children and adolescents (≤18 years) with histologically confirmed sarcomas, including Ewing sarcoma, osteosarcoma, and rhabdomyosarcoma, were consecutively enrolled at diagnosis or before initiation of systemic therapy according to institutional treatment protocols of Fatima Jinna Medical Institution. Patients with concurrent malignancies or severe comorbidities affecting blood sampling were excluded. The sample size was determined based on feasibility and anticipated recruitment during the study period, consistent with exploratory longitudinal biomarker studies in pediatric oncology [28,29]. Treatment followed contemporary risk-adapted institutional protocols.

**Figure 1:**
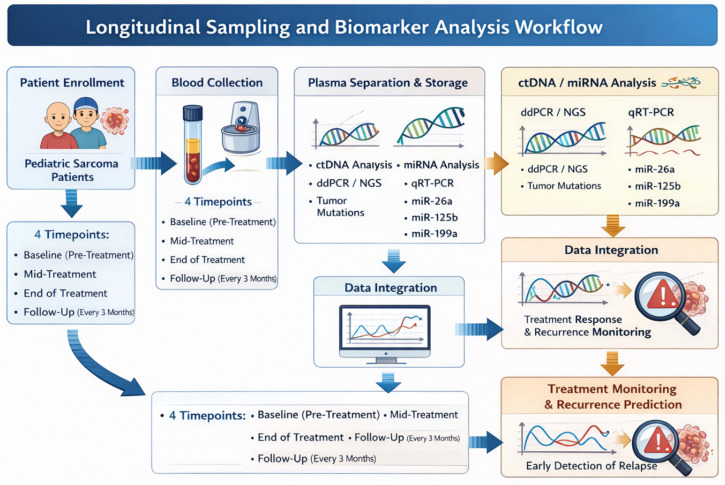
Longitudinal sampling and biomarker analysis workflow in pediatric sarcoma patients. Peripheral blood (5–10 mL) was collected at baseline (T0), during treatment (T1), end of therapy (T2), and follow-up (T3^+^). Plasma was processed within two hours and stored at −80°C. Circulating tumour DNA was assessed using droplet digital PCR or next-generation sequencing, while circulating microRNAs were quantified by ddPCR or qRT-PCR. Biomarker data were integrated with clinical and radiologic findings to evaluate treatment response and detect recurrence. (Created with BioRender.com).

Most patients received multi-agent neoadjuvant chemotherapy, commonly including vincristine, doxorubicin, cyclophosphamide or ifosfamide, and etoposide. Surgical resection was performed when feasible, and radiotherapy was administered in selected cases according to tumor type, resectability, margin status, and treatment response. A schematic overview of patient enrollment, longitudinal blood sampling, plasma processing, and ctDNA/miRNA analysis is provided in Fig. 1.

### 2.2 Blood Collection and Plasma Processing

Peripheral blood samples (5–10 mL) were collected in EDTA tubes at baseline (before initiation of therapy), mid-treatment (after completion of cycle 3 of chemotherapy), at the end of treatment (upon completion of the planned chemotherapy regimen), and during follow-up at three-month intervals. Plasma was separated within two hours of collection by centrifugation at 1600× *g* for 10 min at 4°C, followed by a second high-speed centrifugation at 16,000× *g* for 10 min at 4°C to minimise genomic DNA contamination. Plasma aliquots were stored at −80°C until analysis.

### 2.3 Circulating Tumor DNA Analysis

Total plasma cfDNA concentration (ng/mL) was quantified to assess overall circulating DNA burden, whereas mutation-specific ddPCR assays were used to detect tumor-derived ctDNA. The isolation was done using a commercially available circulating cell-free DNA extraction kit (Cat. No. 55114, Qiagen, Hilden, Germany) according to the manufacturer’s instructions. ddPCR was performed using mutation-specific assays targeting tumor-associated genetic alterations relevant to pediatric sarcoma subtypes.

ddPCR assays were selected based on tumor molecular characterization performed at diagnosis. For Ewing sarcoma, assays targeted EWSR1-related rearrangements (fusion-informed targets when available). For rhabdomyosarcoma and osteosarcoma, assays targeted subtype-relevant alterations identified from tumor tissue (somatic variants/structural changes where applicable). Primer sequences, amplicon information, and annealing temperatures were selected based on the previous literature to ensure full reproducibility [31,32,33]. Each ddPCR reaction was prepared in a total volume of 20 μL containing 10 μL of 2× ddPCR Supermix for Probes (Cat. No. 1863024, Bio-Rad, Hercules, CA, USA), 900 nM of each primer, 250 nM hydrolysis probe, and 5–10 ng of ctDNA template. Droplets were generated using the QX200 Droplet Generator (1864002, Bio-Rad, Hercules, USA), followed by PCR amplification under the following cycling conditions: initial denaturation at 95°C for 10 min; 40 cycles of 94°C for 30 s and 58°C for 60 s; and a final enzyme deactivation step at 98°C for 10 min.

Each sample was analyzed in technical duplicate. Runs were accepted only when >10,000 accepted droplets were generated per reaction and droplet clusters showed clear separation. No-template controls were included in every run to monitor contamination. Where available, mutation-negative plasma controls were used to confirm assay specificity, and tumor DNA with known alterations served as positive controls. Fluorescence thresholds were defined based on the signal distribution of negative droplets using QuantaSoft Analysis Pro Software (Version 1.7.4 or 1.7.5, Bio-Rad Laboratories, Hercules, CA, USA) according to the manufacturer’s guidelines.

### 2.4 Circulating microRNA (miRNA) Analysis

Total RNA concentration (ng/μL) was measured prior to cDNA synthesis; specific miRNA targets (miR-21, miR-26a, miR-125b, miR-199a) were subsequently quantified using ddPCR and qRT-PCR assays. Plasma microRNAs were isolated from 300 μL of plasma using the Maxwell RSC miRNA Plasma and Serum Kit (Cat. No. AS1680, Promega, Madison, WI, USA) on the Maxwell RSC Instrument, in accordance with the manufacturer’s instructions. Total RNA concentration was quantified using the Quantus™ Fluorometer in combination with the QuantiFluor™ RNA System. Complementary DNA (cDNA) was synthesized using the TaqMan™ Advanced miRNA cDNA Synthesis Kit (Cat. No. A28007, Thermo Fisher Scientific, Waltham, MA, USA). Selected miRNAs of interest (miR-21, miR-26a, miR-125b, and miR-199a) [31,32,33] were quantified using droplet digital PCR (ddPCR) on the QX200 Droplet Digital PCR System (Model: QX200; Bio-Rad Laboratories). Each ddPCR reaction was prepared in a total volume of 20 μL containing 10 μL of 2× ddPCR Supermix for Probes (No dUTP) (Cat. No. 1863024, Bio-Rad Laboratories), miRNA-specific primers (900 nM) and probes (250 nM), and 2 μL of cDNA template. Thermal cycling conditions were 95°C for 10 min, followed by 40 cycles of 94°C for 30 s and 60°C for 60 s, with a final step at 98°C for 10 min. Fluorescence thresholds were determined based on the distribution of negative droplets using QuantaSoft Analysis Software (Version 1.7.4, Bio-Rad Laboratories) according to the manufacturer’s guidelines. The limit of detection (LOD) was defined as the lowest concentration producing detectable positive droplets above the negative control background. For comparison, miRNA expression was also measured using qRT-PCR, with Ct values determined automatically based on the instrument threshold set above baseline fluorescence.

In parallel, individual miRNA targets were quantified using qRT-PCR for comparative analysis. For qRT-PCR assays, miRNA expression levels were normalised to miR-16, which was selected as an endogenous control based on its reported stability in plasma samples. All reactions were performed in technical duplicates and included no-template controls and positive controls to ensure assay specificity, reproducibility, and analytical reliability across all sampling time points.

## 3 Statistical Analysis

Descriptive statistics summarized patient demographics, tumor characteristics, and biomarker levels. Paired comparisons between baseline and end-treatment biomarker levels were conducted using the Wilcoxon signed-rank test. Survival analyses were performed using Kaplan–Meier curves (KMC), and associations with recurrence were evaluated using multivariable regression models. Overall survival status at last follow-up (alive vs. deceased) was assessed using multivariable logistic regression and is reported as odds ratios (OR) with 95% confidence intervals. Forest plots were generated to present pooled effect estimates with 95% confidence intervals (CIs). Heterogeneity among studies was assessed using the I^2^ statistic and Cochran’s Q test, and either fixed- or random-effects models were applied accordingly. Receiver operating characteristic (ROC) curves also assessed biomarker sensitivity and specificity for recurrence detection. Statistical analyses were performed using SPSS version 26 (IBM Corp., Armonk, NY, USA), with *p* < 0.05 considered significant.

## 4 Results

A total of 100 pediatric and adolescent patients (≤18 years) with histologically confirmed sarcomas were included in the analysis. The mean age at diagnosis was 10.50 ± 4.9 years, with a male predominance (59%). The most common tumor subtype was Ewing sarcoma (49%), followed by rhabdomyosarcoma (27%) and osteosarcoma (24%). Most patients presented with early to intermediate disease, with 28% and 47% classified as stage I and stage II, respectively, while 15% and 10% had stage III and stage IV disease (Table 1). At baseline, the mean plasma volume obtained was 7.24 ± 1.32 mL. The mean baseline circulating cell-free DNA concentration was 31.12 (16.96–59.00) ng/mL, while the mean baseline circulating microRNA (miRNA) level was 5.02 (2.55–9.30) ng/μL (Table 1).

**Table 1:** Patient demographics, tumor features, and circulating biomarkers (*n* = 100).

Variable	Value
**Age (years) Mean + SD**	10.50 ± 4.95
**Weight (kg) Mean + SD**	44.23 ± 15.20
**Height (cm) Mean + SD**	115.98 ± 49.59
**Baseline Plasma Volume (mL) Mean + SD**	7.24 ± 1.32
**Baseline Circulating cfDNA (ng/mL) Median (IQR)**	31.12 (16.96–59.00)
**Baseline Circulating miRNA (ng/μL) Median (IQR)**	5.02 (2.55–9.30)
**Sex**	
Male, n (%)	59 (59.0%)
Female, n (%)	41 (41.0%)
**Tumor Type**	
Ewing sarcoma, n (%)	49 (49.0%)
Osteosarcoma, n (%)	24 (24.0%)
Rhabdomyosarcoma, n (%)	27 (27.0%)
**Tumor Stage**	
Stage I, n (%)	28 (28.0%)
Stage II, n (%)	47 (47.0%)
Stage III, n (%)	15 (15.0%)
Stage IV, n (%)	10 (10.0%)

Data are presented as mean ± standard deviation (SD), Median (IQR) and number (%). IQR = interquartile range (25th–75th percentile), cfDNA = circulating free DNA, miRNA = microRNA, n: number.

### 4.1 Changes in Circulating Biomarkers during Treatment

Paired analyses demonstrated a significant reduction in circulating biomarkers from baseline to the end of treatment. Median cfDNA concentration decreased from 31.12 ng/mL (IQR: 16.96–59.00) at baseline to 21.60 ng/mL (IQR: 10.12–38.00) at the end of treatment (*p* < 0.001). Similarly, median circulating miRNA concentration declined from 5.02 ng/μL (IQR: 2.55–9.30) to 3.35 ng/μL (IQR: 2.00–5.00) over the treatment course (*p* < 0.001) (Table 2). These findings demonstrate a significant reduction in circulating biomarkers during therapy, consistent with treatment-related tumor burden reduction.

**Table 2:** Comparison of baseline and end-of-treatment circulating biomarkers.

Biomarker	Baseline, Median (IQR)	End of Treatment, Median (IQR)	*p* Value*
**Circulating ctDNA (ng/mL)**	31.12 (16.96–59.00)	21.60 (10.12–38.00)	<0.001
**Circulating miRNA (ng/μL)**	5.02 (2.55–9.30)	3.35 (2.00–5.00)	<0.001

*Paired comparisons were performed using the Wilcoxon signed-rank test. IQR = interquartile range (25th–75th percentile). A *p*-value < 0.05 (*) was considered statistically significant.

### 4.2 Association between End-of-Treatment Biomarkers and Treatment Response

Correlation analyses revealed no statistically significant associations between end-of-treatment circulating biomarkers and clinical or radiologic response. Clinical response status showed weak negative correlations with radiologic response (r = −0.084, *p* = 0.406), end-of-treatment ctDNA (r = −0.121, *p* = 0.230), and end-of-treatment miRNA (r = −0.080, *p* = 0.431). Likewise, radiologic response exhibited weak correlations with end-of-treatment ctDNA (r = −0.173, *p* = 0.086) and miRNA (r = 0.015, *p* = 0.884). End-of-treatment ctDNA and miRNA levels were also weakly correlated (r = 0.031, *p* = 0.761) (Table 3).

**Table 3:** Pearson correlations between end-of-treatment circulating biomarkers and treatment response (n = 100).

Variable	Radiologic Response (R/N)	End-of-Treatment cfDNA (ng/mL)	End-of-Treatment miRNA (ng/μL)
**Clinical Response (R/N)**	−0.084 (*p* = 0.406)	−0.121 (*p* = 0.230)	−0.080 (*p* = 0.431)
**Radiologic Response (R/N)**	—	−0.173 (*p* = 0.086)	0.015 (*p* = 0.884)
**End-of-Treatment cfDNA**	—	—	0.031 (*p* = 0.761)

Values represent Pearson correlation coefficients (*r*) with corresponding *p* values. Clinical and radiologic responses were coded as responder/non-responder (R/N).

End-of-treatment ctDNA and miRNA levels were comparable across the three sarcoma subtypes. Median ctDNA values were 21.5 (11–29) in Ewing sarcoma, 19.5 (13–31) in osteosarcoma, and 19.5 (10–32) in rhabdomyosarcoma, with no statistically significant difference observed (*p* = 0.672). Similarly, miRNA expression levels did not differ significantly among tumor types, with median values of 2.9 (2.3–3.9), 2.7 (2.1–4.0), and 3.1 (2.5–3.8), respectively (*p* = 0.192). These findings indicate that end-treatment biomarker levels were not tumor-type specific in this cohort (Table 4).

**Table 4:** Comparison of tumor types with end-of-treatment ctDNA and miRNA levels.

Variable	Ewing Sarcoma (n = 49)	Osteosarcoma (n = 24)	Rhabdomyosarcoma (n = 27)	*p*-Value*
**End-Treatment cfDNA, Median (IQR)**	21.5 (11–29)	19.5 (13–31)	19.5 (10–32)	0.672
**End-Treatment miRNA, Median (IQR)**	2.9 (2.3–3.9)	2.7 (2.1–4.0)	3.1 (2.5–3.8)	0.192

*p*-values (*) were calculated using the Kruskal–Wallis test for comparison among the three tumor types. IQR = interquartile range (25th–75th percentile). ctDNA = circulating tumor DNA.

### 4.3 Overall Survival According to Clinical and Radiologic Response

**Figure 2:**
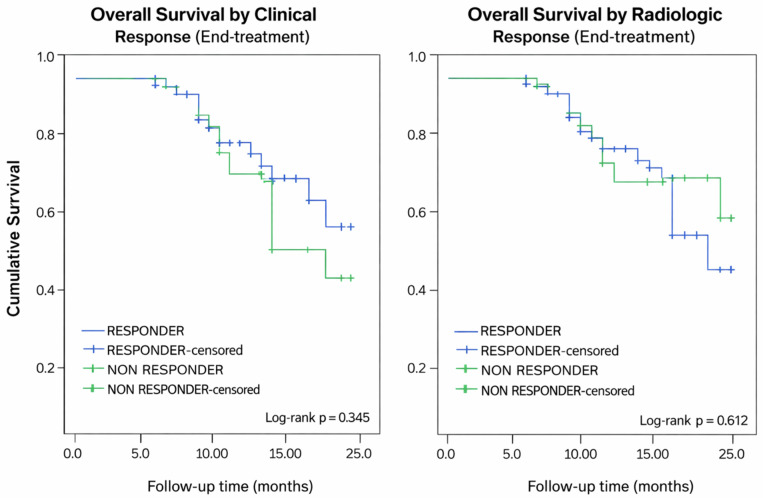
Mean Survival Time by Clinical and Radiologic Response Status. Kaplan–Meier analysis demonstrated no significant difference in overall survival between end-of-treatment clinical or radiologic response groups. Clinical responders showed slightly longer mean survival than non-responders (20.49 ± 0.70 vs. 18.68 ± 1.18 months; log-rank *p* = 0.345). Similarly, radiologic responders and non-responders had comparable survival outcomes (19.84 ± 0.76 vs. 20.51 ± 1.02 months; log-rank *p* = 0.612).

KMC analysis showed no significant difference in overall survival (OS) between end-of-treatment clinical or radiologic response groups. Mean survival time was slightly longer in clinical responders compared with non-responders (20.49 ± 0.70 months vs. 18.68 ± 1.18 months), although this difference was not statistically significant (log-rank *p* = 0.345). Similarly, radiologic responders and non-responders showed comparable survival outcomes (19.84 ± 0.76 months vs. 20.51 ± 1.02 months; log-rank *p* = 0.612) (Fig. 2). This indicates that end-of-treatment response status alone did not independently predict OS during the two-year follow-up period.

### 4.4 Predictors of Outcome in Multivariable Analysis

Multivariable logistic regression analysis identified recurrence status that was strongly associated with overall survival, reflecting the established clinical relationship between relapse and mortality; however, circulating biomarker levels were not independently predictive. Patients who experienced disease recurrence had a significantly lower likelihood of survival (OR = 0.04, 95% CI: 0.01–0.15; *p* < 0.001). In contrast, baseline and end-of-treatment ctDNA and miRNA levels were not significantly associated with outcome. Age, sex, tumor type and stage also showed no significant independent associations (Table 5).

**Figure 3:**
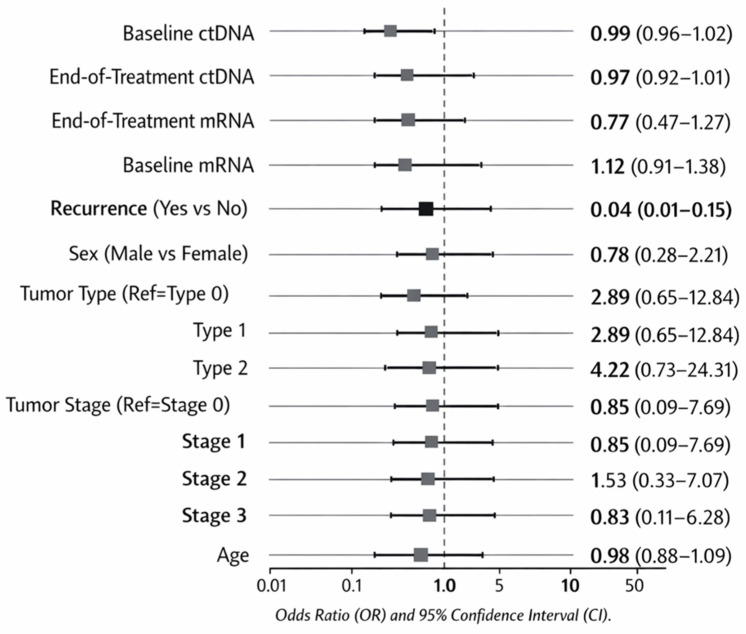
Forest plot of odds ratios (OR) with 95% confidence intervals for factors associated with the outcome. The forest plot showed that recurrence status had the strongest and only statistically significant association with survival (OR = 0.04; 95% CI: 0.01–0.15). In contrast, ctDNA, mRNA levels, and other clinical variables demonstrated odds ratios close to unity with wide confidence intervals crossing 1.0, indicating no statistically significant relationship with survival outcomes in this cohort.

The forest plot showed that recurrence status had the largest and only statistically significant effect among the evaluated variables. In contrast, biomarker levels and clinical characteristics showed wide confidence intervals that crossed unity (Fig. 3).

**Table 5:** Multivariable logistic regression analysis of factors associated with OS (n = 100)*.

Variable	B	SE	Wald	*p* Value	OR	95% CI
**Baseline Circulating ctDNA (ng/mL)**	−0.011	0.016	0.538	0.463	0.99	0.96–1.02
**End-of-Treatment Circulating ctDNA (ng/mL)**	−0.036	0.023	2.539	0.111	0.97	0.92–1.01
**End-of-Treatment Circulating miRNA (ng/mL)**	−0.263	0.256	1.061	0.303	0.77	0.47–1.27
**Baseline Circulating miRNA (ng/μL)**	0.113	0.107	1.114	0.291	1.12	0.91–1.38
**Recurrence (Yes vs. No)**	−3.239	0.674	23.082	<0.001	0.04	0.01–0.15
**Sex (Male vs. Female)**	−0.248	0.532	0.217	0.641	0.78	0.28–2.21
**Tumor type**	—	—	2.742	0.254	—	—
Ewing sarcoma	1.059	0.762	1.934	0.164	2.89	0.65–12.84
Osteosarcoma	1.440	0.893	2.600	0.107	4.22	0.73–24.31
**Tumor stage**	—	—	1.224	0.747	—	—
Stage I	−0.164	1.125	0.021	0.884	0.85	0.09–7.69
Stage II	0.422	0.782	0.291	0.589	1.53	0.33–7.07
Stage III	−0.183	1.031	0.032	0.859	0.83	0.11–6.28
**Age (years)**	−0.020	0.052	0.149	0.700	0.98	0.88–1.09

*OS: Overall survival (event vs. no event) was used as the dependent variable. Tumour type and stage were entered as categorical variables. Odds ratios (OR) and 95% confidence intervals (CI) are shown. B, Regression Coefficient; SE, Standard Error.

## 5 Discussion

In this prospective cohort of pediatric patients with sarcomas (n = 100), circulating biomarkers showed a clear treatment-associated decline, with both ctDNA and circulating miRNA concentration significantly lower at end of treatment compared with baseline (both *p* < 0.001). This consistent downward trend supports the concept that circulating tumor–derived signals can reflect global tumor burden and response to therapy [34], even in pediatric cancers where mutational burden and tumor biology differ from adult malignancies and where routine ctDNA integration remains limited in many centers [35]. Importantly, the observed decline across two independent analyte classes (ctDNA and miRNA) strengthens biological plausibility and supports the use of multi-analyte liquid biopsy strategies in pediatric sarcoma monitoring [36]. Despite this significant reduction in biomarker levels, end-treatment ctDNA and miRNA were not significantly correlated with clinical or radiologic response, and correlations between response metrics and biomarker endpoints were weak and statistically insignificant. This pattern is consistent with broader liquid biopsy experience: single “snapshot” measurements may fail to capture meaningful residual disease biology, particularly when disease burden is low, heterogeneous, or when response assessments are influenced by timing, imaging sensitivity, and treatment-related changes [37]. In pediatric sarcomas specifically, prior studies showed that ctDNA detectability and prognostic impact can vary by subtype, disease burden, and assay design (e.g., fusion-based vs. broader panels), suggesting that dynamic longitudinal change may be more informative than end-treatment values alone [38,39].

A key finding in the current cohort was that recurrence status emerged as the only significant independent predictor in the multivariable model, whereas baseline and end-treatment ctDNA/miRNA did not independently predict the modeled outcome. This result should be interpreted cautiously because recurrence is often linked to downstream outcomes by definition and may reflect temporal overlap between predictor and endpoint. Nevertheless, the finding emphasizes a central clinical opportunity for liquid biopsy in pediatric sarcoma: rather than expecting end-of-therapy levels to mirror response categories, the strongest value may lie in serial surveillance, where rising ctDNA (or miRNA) could flag molecular relapse earlier than conventional detection. This concept is supported by studies in pediatric sarcomas linking ctDNA detection to poorer clinical outcomes, as well as by methodological advances that allow reliable identification of sarcoma-associated genomic alterations, including characteristic translocations and copy-number changes [40].

From a translational perspective, the performance of pediatric sarcoma liquid biopsies depends heavily on assay strategy. Ewing sarcoma, for example, offers a biologically tractable target through tumor-defining fusions, and fusion-informed approaches have shown promise as highly sensitive plasma markers in Ewing sarcoma [41]. Likewise, osteosarcoma studies support ctDNA detection feasibility, but emphasize challenges related to genomic complexity and heterogeneity, reinforcing the need for optimized panels and longitudinal interpretation [42]. In parallel, circulating miRNAs have demonstrated clinical relevance across cancers and may complement ctDNA by capturing tumor-associated signaling, host response, or disease biology not fully represented by mutation-only assays [35]. Advances in pediatric translational infrastructure, including genomically annotated models and precision oncology registries, further support validation of biomarker-driven monitoring and risk stratification strategies [43].

Future studies should also assess combined ctDNA and microRNA approaches and determine whether biomarker kinetics enhance relapse prediction beyond conventional imaging. In summary, our results support ctDNA and circulating miRNA as therapy-responsive biomarkers in pediatric sarcoma, but also demonstrate that end-treatment measurements and simple correlations with response status may be insufficient. The next step is to focus on serial kinetics, subtype-specific assay optimization (e.g., fusion-aware designs for Ewing sarcoma), and prospective multicenter validation embedded in pediatric clinical trial settings.

## 6 Limitation

The study was conducted at a single center with a relatively limited sample size, potentially restricting statistical power for subgroup analyses. Second, healthy pediatric controls were not included due to ethical constraints, limiting the interpretation of baseline biomarker ranges. Third, pathological response data were not systematically collected, precluding correlation between biomarker kinetics and histologic response. Finally, survival modeling was limited by follow-up duration and event numbers.

## 7 Conclusion

In this prospective cohort study of pediatric sarcoma patients, tumor-derived circulating biomarkers exhibited distinct treatment-related dynamics. Both circulating tumor DNA and microRNA levels declined significantly during therapy, reflecting tumor burden reduction and therapeutic response at the population level. However, biomarker levels measured at treatment completion did not independently predict clinical response, radiologic findings, or survival, highlighting the limitations of single-time point liquid biopsy assessments. These results underscore the difference between biomarker responsiveness and prognostic value and suggest that liquid biopsy may be most useful for longitudinal disease monitoring rather than endpoint prediction. The strong association between recurrence and outcome further emphasizes the need for surveillance tools capable of detecting molecular relapse earlier than conventional methods. Collectively, these findings support the feasibility of incorporating ctDNA and circulating microRNA analysis into pediatric sarcoma monitoring strategies while reinforcing the importance of prospective, multicenter studies to validate serial biomarker kinetics, optimize subtype-specific assays, and evaluate combined multi-analyte approaches for improving disease surveillance and personalized management in pediatric sarcoma care.

## Data Availability

The datasets generated during and/or analysed during the current study are available from the corresponding author [Maher Kurdi] on reasonable request.
